# Urticaria and angioedema

**DOI:** 10.1186/1710-1492-7-S1-S9

**Published:** 2011-11-10

**Authors:** Amin Kanani, Robert Schellenberg, Richard Warrington

**Affiliations:** 1 Division of Allergy and Immunology, Department of Medicine, University of British Columbia, Vancouver, British Columbia, Canada; 2 University of Manitoba, Winnipeg, Manitoba, Canada

## Abstract

Urticaria (hives) is a common disorder that often presents with angioedema (swelling that occurs beneath the skin). It is generally classified as acute, chronic or physical. Second-generation, non-sedating H1-receptor antihistamines represent the mainstay of therapy for both acute and chronic urticaria. Angioedema can occur in the absence of urticaria, with angiotensin-converting enzyme (ACE) inhibitor-induced angioedema and idiopathic angioedema being the more common causes. Rarer causes are hereditary angioedema (HAE) or acquired angioedema (AAE). Although the angioedema associated with these disorders is often self-limited, laryngeal involvement can lead to fatal asphyxiation in some cases. The management of HAE and AAE involves both prophylactic strategies to prevent attacks of angioedema (i.e., trigger avoidance, attenuated androgens, tranexamic acid, and plasma-derived C1 inhibitor replacement therapy) as well as pharmacological interventions for the treatment of acute attacks (i.e., C1 inhibitor replacement therapy, ecallantide and icatibant). In this article, the authors review the causes, diagnosis and management of urticaria (with or without angioedema) as well as the work-up and management of isolated angioedema, which vary considerably from that of angioedema that occurs in the presence of urticaria.

## Introduction

**Figure 1:**
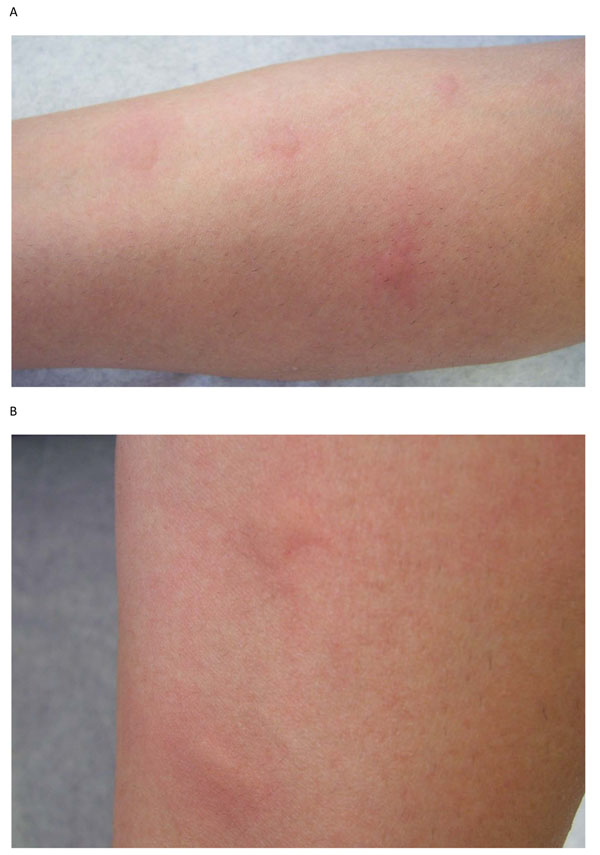
Urticaria (hives).

Urticaria (hives) is a common disorder, occurring in 15-25% of individuals at some point in life [1,2]. It is characterized by recurrent, pruritic (itchy), pink-to-red edematous (swollen) lesions that often have pale centers (wheals) (see Figure 1). The lesions can range in size from a few millimeters to several centimeters in diameter, and are often transient, lasting for less than 48 hours [1-4]. Approximately 40% of patients with urticaria also experience angioedema (swelling that occurs beneath the skin) [1].

Mast cells are the primary effector cells in urticaria and in many cases of angioedema. These cells are widely distributed in the skin, mucosa, and other areas of the body, and have high-affinity immunoglobulin E (IgE) receptors. Mast cell degranulation leads to the rapid release of various inflammatory mediators, such as histamine, leukotrienes and prostaglandins, which, in turn, cause vasodilation and leakage of plasma in and below the skin. There is also a more delayed (4–8 hour) secretion of inflammatory cytokines (e.g., tumor necrosis factor, interleukin 4 and 5) that potentially leads to further inflammatory responses and longer-lasting lesions [1].

**Figure 2:**
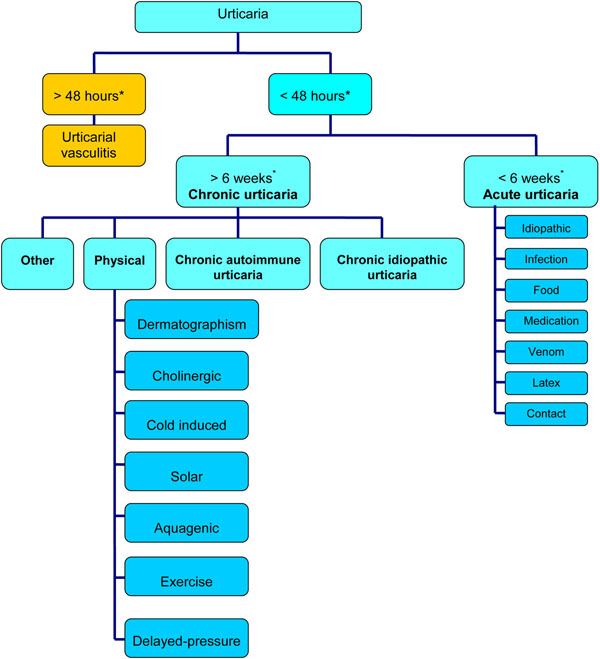
**Classification of urticaria: overview.** *The 48-hour cut-off refers to individual lesions, while the 6-week cut-off refers to the condition as a whole.

Urticaria is generally classified as acute, chronic, or physical, depending on the duration of symptoms and the presence or absence of inducing stimuli (see Figure 2). Acute urticaria refers to lesions that occur for less than 6 weeks, and chronic urticaria to lesions that occur for more than 6 weeks; it is usually assumed that the lesions are present most days of the week [5]. Most cases of urticaria are acute; approximately 30% go on to become chronic. Physical urticaria represents a distinct subgroup of chronic urticaria that is induced by external physical stimuli, such as scratching (dermatographism, a common form of physical urticaria), cold, heat, sunlight and pressure.

Although acute urticaria can generally be easily managed and is associated with a good prognosis, chronic, severe urticaria is often associated with significant morbidity and a diminished quality of life [6]. Physical urticaria also tends to be more severe and long-lasting, and is often difficult to treat [1,3].

The first part of this article will focus on the causes, diagnosis and management of the most common types of urticaria (with or without angioedema). As mentioned earlier, angioedema commonly occurs with urticaria, and evaluation and management are similar to those for urticaria. The latter section will review the work-up and management of isolated angioedema, which varies considerably from the diagnosis and treatment of angioedema that occurs in the presence of urticaria.

## Classification and etiology

### Chronic urticaria

Chronic urticaria is more common in adults, and affects women more frequently than men [1,4]. In general, chronic urticaria is classified as either chronic autoimmune urticaria or chronic idiopathic urticaria (see Figure 2) [4,5]. In chronic autoimmune urticaria, circulating immunoglobulin G (IgG) autoantibodies react to the alpha subunit of the high-affinity IgE receptor on dermal mast cells and basophils, leading to chronic stimulation of these cells and the release of histamine and other inflammatory mediators which cause urticaria and angioedema [2,5]. Chronic autoimmune urticaria is also associated with antithyroid antibodies in approximately 27% of cases, as well as other autoimmune conditions such as vitiligo (a chronic disorder that causes depigmentation of patches of skin) and rheumatoid arthritis [1,2,5]. It has also been proposed that *Helicobacter pylori* (*H. pylori*), which has an immunogenic cell envelope, may play an indirect role in the etiology of chronic autoimmune urticaria by reducing immune tolerance and inducing autoantibody formation. However, it is important to note that the limited number of studies conducted in this area have yielded conflicting results [7,8].

Patients with chronic idiopathic urticaria do not have evidence of autoimmunity. In this form of urticaria, there appears to be persistent activation of mast cells, but the mechanism of mast cell triggering is unknown. Although rare, chronic urticaria may also be a manifestation of a systemic illness [1,3].

### Acute urticaria

The most common causes of acute urticaria (with or without angioedema) are medications, foods, viral infections, parasitic infections, insect venom, and contact allergens, particularly latex hypersensitivity. Medications known to commonly cause urticaria ± angioedema include antibiotics (particularly penicillins, and sulfonamides), non-steroidal anti-inflammatory drugs (NSAID), acetylsalicylic acid (ASA), opiates and narcotics. The predominant foods that cause urticaria are milk, eggs, peanuts, tree nuts, fish, and shellfish. In approximately 50% of patients with acute urticaria, the cause is unknown (idiopathic urticaria) [1,2,9].

### Physical urticaria

As mentioned earlier, physical urticaria is triggered by a physical stimulus. The most common physical urticaria is dermatographism (also known a “skin writing”), in which lesions are created or “written” on the skin by stroking or scratching the skin. Cholinergic urticaria is also common and results from a rise in basal body temperature that occurs following physical exertion or exposure to heat. Other physical stimuli which can trigger urticaria include exposure to cold (cold-induced urticaria), ultraviolet light (solar urticaria), water (aquagenic urticaria) and exercise. The lesions produced by these physical stimuli are typically localized to the stimulated area and often resolve within 2 hours. However, some patients may experience delayed-pressure urticaria which, as the name implies, comes on slowly after pressure has been applied (i.e., 30 minutes to 12 hours) and can last several hours or even days. Examples of sites typically affected include the waistline (after the wearing of tight-fitting pants) and the area of the ankles or calves that makes contact with the elastic band of socks [1-3,5].

## Diagnosis

The diagnosis of urticaria, with or without angioedema, is based primarily on a thorough clinical history and physical examination. Based on the history and physical exam, diagnostic tests may also be considered to help confirm a diagnosis of acute, chronic or physical urticaria.

### History and physical examination

The history and physical examination should include detailed information regarding: the frequency, timing, duration and pattern of recurrence of lesions; the shape, size, site and distribution of lesions; potential triggers (e.g., food, medications, physical stimuli, infections, insect stings); response to previous therapies used; and a personal or family history of atopy [1,3].

Many conditions can easily be confused with urticaria, particularly urticarial vasculitis and systemic mastocytosis (see Table 1 for conditions that need to be considered in the differential diagnosis of urticaria). In urticarial vasculitis, the lesions are usually painful rather than pruritic, last longer than 48 hours, and leave bruises or discoloration on the skin [1,10]. Systemic mastocytosis (also called systemic mast cell disease) is a rare condition that involves the internal organs, in addition to the skin. In this disorder, atypical mast cells collect in various tissues that can affect the liver, spleen, lymph nodes, bone marrow and other organs [1,9].

**Table 1:** Conditions to consider in the differential diagnosis of urticaria.

Urticarial vasculitis	• Lesions are usually painful (rather than pruritic), last >48 hours, and leave discoloration on the skin
Systemic mastocytosis	• Rare condition that involves the internal organs (liver, spleen, lymph nodes, bone marrow), in addition to the skin

Atopic dermatitis	• Chronic, highly pruritic inflammatory skin disease • Clinical manifestations vary with age

Bullous pemphigoid	• Chronic, autoimmune, blistering skin disease

Erythema multiforme	• Acute, self-limited, skin condition • Considered to be a type IV hypersensitivity reaction to certain infections, medications, and other various triggers

Familial cold autoinflammatory syndrome	• Rare, inherited inflammatory disorder characterized by recurrent episodes of rash, fever/chills, joint pain, and other signs/symptoms of systemic inflammation triggered by exposure to cooling temperatures • Onset usually occurs during infancy and early childhood and persists throughout the patient’s life

Fixed drug eruptions	• Lesions occur from exposure to a particular medication and occur at the same site upon re-exposure to the offending medication • Lesions usually blister and leave residual pigmentation

Subacute cutaneous lupus erythematosus	• A non-scarring, photosensitive skin condition • May occur in patients with systemic lupus erythematosus (SLE) and Sjögren syndrome

Pruritic urticarial papules and plaques of pregnancy	• Benign skin condition that usually arises late in the third trimester of a first pregnancy

Muckle-Wells syndrome	• Rare genetic disease that causes hearing loss and recurrent hives • May lead to amyloidosis

Schnitzler's syndrome with monoclonal IgG kappa gammopathy	• Rare disease characterized by chronic, non-pruritic hives, periodic fever, bone and joint pain, swollen lymph glands and an enlarged spleen and liver

### Diagnostic tests

Skin prick tests (SPTs) and serum-specific IgE tests may help confirm a diagnosis of acute urticaria resulting from allergic or IgE-mediated (type I) reactions to common food allergens, latex hypersensitivity, stinging insect hypersensitivity and certain antibiotics These tests are best performed by allergists with experience in interpreting test results in the appropriate clinical context.

Certain diagnostic tests and assessments can be helpful in the diagnosis and differential diagnosis of chronic urticaria, including: a complete blood count (CBC), serum protein electrophoresis (SPE), the autologous serum skin test (ASST), the basophil activation test, thyroid autoantibodies, *H. pylori*, antinuclear antibodies (ANA), and erythrocyte sedimentation rate (ESR). SPE can be used to identify increases in IgG and, therefore, may help confirm the diagnosis of chronic autoimmune disease. The presence of thyroid autoantibodies is also suggestive of chronic autoimmune urticaria. An elevated ESR or ANA is often indicative of an underlying systemic condition, such chronic infection or vasculitis [2].

The ASST is currently one of the most useful tests for confirming a diagnosis of chronic autoimmune urticaria (sensitivity: 65–81%; specificity: 71–78%) [11]. It involves intradermal injection of the patient’s own serum (collected while the patient is symptomatic) into uninvolved skin. A positive wheal and flare reaction is considered indicative of circulating autoantibodies to the high-affinity IgE receptor on mast cells. However, it should be noted that the ASST is not widely available and is often poorly tolerated by younger children due to the discomfort associated with the intradermal injections [3]. Since basophils are also involved in chronic urticaria, the basophil activation test (the quantification of basophil activation by flow cytometry) may be useful for screening for the autoimmune form of the disease. However, further confirmatory studies are needed before this test is widely accepted as a diagnostic tool [2].

Challenge testing, which reproduces exposure to a suspected stimuli in a supervised clinical environment, is often indicated to confirm a diagnosis of physical urticaria. Cold-induced urticaria can usually be confirmed using the ice cube test (i.e., placing an ice cube in a sealed plastic bag over the forearm for 5-10 min). Dermatographism can be confirmed by lightly scratching the skin, and aquagenic urticaria can be identified by immersion of a body part into warm water or through the application of warm compresses. Hot bath testing can help identify cholinergic urticaria, and the application of weight/pressure to the patient’s thigh is helpful in the diagnosis of delayed-pressure urticaria [1,2].

## Treatment

**Figure 3:**
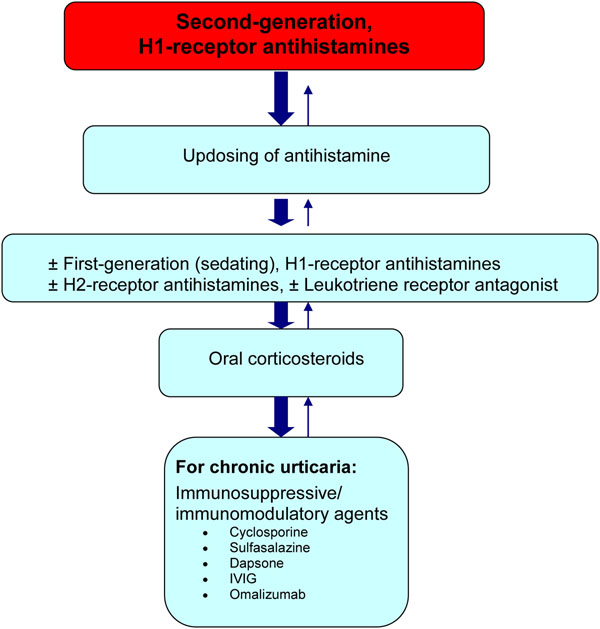
**A simplified, stepwise algorithm for the treatment of urticaria.*** IVIG: intravenous immunoglobulin G*

Strategies for the management of acute urticaria include avoidance measures, antihistamines and corticosteroids. For urticaria, antihistamines are the mainstay of therapy. Corticosteroids and various immunomodulatory/ immunosuppressive therapies may also be used for more severe cases, or for those patients who experience a poor response to antihistamines (see Figure 3).

### Avoidance

For some patients with acute urticaria, a specific trigger can be identified (e.g., food, medication, latex, insect venom), and avoidance of the offending agent can be an effective management approach. Patients should be provided with clear, written instructions on appropriate avoidance strategies [2,3].

### Antihistamines

Second-generation, non-sedating, H1-receptor antihistamines (e.g., fexofenadine, desloratadine, loratadine, cetirizine) are the mainstay of therapy for urticaria. These agents have been shown to be significantly more effective than placebo for the treatment of both acute and chronic urticaria [4]. First-generation, sedating antihistamines may be used as adjunctive therapy in those patients who have difficulty sleeping due to nocturnal symptoms [1-3]. Table 2 provides a list of commonly used second- and first-generation antihistamines and their recommended dosing regimens. Since 15% of histamine receptors in the skin are H2-type receptors, H2-receptor antihistamines, such as cimetidine, ranitidine and nizatidine, may also be helpful in some patients with urticaria. However, these agents should not be used as monotherapy as they have limited effects on pruritus [2].

**Table 2:** Antihistamines commonly used and indicated for the treatment of urticaria.

	Usual adult dose	Usual pediatric dose
**Second-generation H1-receptor antihistamines (first-line therapy)**		

Cetirizine (Reactine)	10-40 mg daily	5-10 mL (1-2 teaspoons) daily (children’s formulation)
Desloratadine (Aerius)	5-20 mg daily	2.5-5 mL (0.5-1.0 teaspoon) daily (children’s formulation)
Fexofenadine (Allegra)	120-480 mg daily	Not currently indicated for children under 12 years of age
Loratadine (Claritin)	10-40 mg daily	5-10 mL (1-2 teaspoons) daily (children’s formulation)

**First-generation H1-receptor antihistamines (best used as adjunctive therapy for patients with nocturnal symptoms)**		

Hydroxyzine (Atarax)	25-50 mg, three to four times daily	Children < 6 years: 30 to 100 mg daily in divided doses
Diphenhydramine (Benadryl)	25-50 mg, every -6 hours	2.5-20 mL (0.5-4 teaspoons) every 4 to 6 hours (depending on age/weight)
Cyproheptadine (Periactin)	4-20 mg daily	2-4 mg, two to three times daily (depending on age/weight)
Chlorpheniramine (Chor-Tripolon)	4 mg every 4–6 hours	1 mg every 4–6 hours
Clemastine (Tavist-1)	1.34 - 2.68 mg, two to three times daily	1.34 mg, once or twice daily

Antihistamine efficacy is often patient specific and, therefore, more than one antihistamine should be tried before assuming therapeutic failure with these agents. Also, antihistamines are most effective if taken daily, rather than on an as-needed basis. If symptoms are controlled with standard antihistamine doses, it is reasonable to continue treatment for several months, occasionally stopping therapy for brief periods to determine whether the urticaria has spontaneously resolved. In patients who do not achieve adequate symptom control at standard doses, it is common practice to increase the antihistamine dose beyond the usual recommended dose. In fact, current European guidelines recommend up to four times the usual recommended dose of antihistamine in patients who symptoms persist with standard therapy [12]. For example, doses of up to 40 mg of cetirizine, 20 mg of desloratadine, and 480 mg of fexofenadine may be used in adults. The efficacy of this approach, however, still requires confirmation in randomized, double-blind controlled trials [2,3].

### Corticosteroids

For some patients with severe urticaria who are inadequately responsive to antihistamines, a brief course of oral corticosteroids (e.g., prednisone, up to 40 mg/day for 7 days) is warranted. However, long-term corticosteroid therapy should be avoided given the well-known side effects associated with prolonged use of corticosteroids and the increased likelihood of developing tolerance to these agents [2,3].

### Immunosuppressive and Immunomodulatory Therapies

Various immunosuppressive or immunomodulatory therapies may provide some benefit for patients with severe, chronic urticaria. Double-blind, randomized controlled trials have found cyclosporine (3–5 mg/kg/day) to be effective in patients with chronic urticaria who do not adequately respond to antihistamines [13,14]. During treatment with cyclosporine, H1-receptor antihistamines should be continued, and blood pressure, renal function and serum levels should be monitored regularly given the significant side effects associated with this form of therapy (e.g., hypertension, renal toxicity).

Case reports and other small clinical trials have also found the following treatments to be effective for select patients with severe, refractory, chronic urticaria: sulfasalazine; the antibacterial, dapsone; the anti-IgE monoclonal antibody, omalizumab; and intravenous immunoglobulin G (IVIG) [4]. However, the efficacy of these agents in the treatment of chronic urticaria needs to be confirmed in large, randomized controlled trials.

### Other therapies

Leukotriene receptor antagonists, such as montelukast (Singulair) or zafirlukast (Accolate), have also been shown to be effective in the treatment of poorly-controlled chronic urticaria [15-17]. However, these agents should only be used as adjuncts to antihistamine therapy as there is little evidence that they are useful as monotherapy. Injectable epinephrine should also be prescribed to patients with a history of severe urticaria and angioedema leading to anaphylaxis (see article on anaphylaxis in this supplement) [1].

## Angioedema (without Urticaria)

### Introduction

Angioedema in the absence of urticaria is rare and should alert the physician to alternative diagnoses, such as hereditary or acquired angioedema, idiopathic angioedema, or angioedema associated with angiotensin-converting enzyme (ACE) inhibitors. Hereditary angioedema (HAE) is a rare autosomal dominant genetic disorder resulting from an inherited deficiency or dysfunction of the C1 inhibitor (a plasma protease inhibitor that regulates several proinflammatory pathways). Two main types of HAE have been defined: type I and type II. Type I HAE is characterized by low C1 inhibitor levels and function (85% of cases) while type II is associated with normal C1 inhibitor levels, but low function (15% of cases). Acquired angioedema (AAE) is another rare C1 inhibitor deficiency syndrome which is most commonly associated with B-cell lymphoproliferative diseases (type 1 AAE). It may also be related to the presence of an autoantibody directed against the C1 inhibitor molecule (type II AAE) [18].

Clinically, HAE and AAE are similar, and are characterized by recurrent episodes of angioedema, without urticaria or pruritus, which most often affect the skin or mucosal tissues of the gastrointestinal and upper respiratory tracts. Although generally benign conditions, laryngeal involvement can rapidly lead to fatal asphyxiation if left untreated. Age of onset and the presence of a familial history are distinguishing features of these conditions (see Table 3). HAE usually presents in late childhood or adolescence in otherwise healthy subjects, and a familial history is present in approximately 75% of cases (with the remaining 25% resulting from spontaneous mutation of the C1 inhibitor gene). In contrast, AAE is not associated with a family history, and usually develops in older patients (fourth decade of life) with an underlying lymphoproliferative or autoimmune disease [18,19].

**Table 3:** Comparison of HAE and AAE.

	Age of onset	Family history	Complement levels			
	
			C1q	C4	C1 inh level	C1 inh function
**HAE** **Type 1** **Type 2**	Early	Yes*	Normal Normal	Low Low	Low Normal/elevated	Low Low

**AAE**	Late	No	Low	Low	Normal or low	Low

*In approximately 25% of patients, no family history is identified; the disorder results from spontaneous mutation of the C1 inhibitor gene.

C1 inh: C1 inhibitor

Although the exact pathogenesis of attacks of HAE and AAE remains unclear, excess production of the potent vasodilatory peptide, bradykinin (which is regulated by the C1 inhibitor), appears to play an important role [20]. It is important to note that histamine and other mast cell mediators that are typical of urticaria and associated angioedema are not directly involved in HAE and AAE, which explains patient lack of response to antihistamines and corticosteroids, and distinguishes these forms of isolated angioedema from that associated with urticaria.

Isolated angioedema also occurs in approximately 0.1% to 6% of individuals using ACE inhibitors. It tends to occur more commonly in ACE-inhibitor users who are female, smokers or of African-American descent. Like HAE and AAE, ACE inhibitor–induced angioedema is bradykinin-mediated. Most cases of angioedema occur in the first week after starting ACE-inhibitor therapy. However, up to one-third of cases occur months to years after initiating the medication [21]. ACE inhibitor-induced angioedema can be life-threatening when it involves the upper airway and, therefore, ACE inhibitors should be discontinued in all individuals with angioedema.

In the majority of cases of isolated angioedema, the cause is not identifiable (idiopathic angioedema). The exact mechanisms of idiopathic angioedema are unclear; however, based on patient response to medication, some cases are believed to be mediated by IgE-independent mechanisms that lead to mast cell activation [22,23]. In most individuals, this condition does not lead to life-threatening angioedema.

## Diagnosis

The diagnosis of HAE and AAE is based upon a suggestive clinical history (i.e., episodic angioedema in the absence of urticaria affecting the skin, gastrointestinal and upper respiratory tracts) and the presence of abnormalities in specific complement proteins. Complement studies that should be ordered for patients with suspected HAE and AAE include: C4 (the natural substrate for C1) level, C1q level, C1 inhibitor antigenic level, and C1 inhibitor functional level [18]. These studies should be performed when the patient is not receiving treatment, since the use of therapeutic interventions for AAE or HAE can alter laboratory results.

In most patients with AAE, C4, C1q, and C1 inhibitor function levels are low (<50% of normal), and C1 inhibitor antigenic levels are low or normal. In type I HAE, C1 inhibitor antigenic and function levels are low (<50% of normal); in type II HAE, C1 inhibitor functional levels are low, but antigenic levels are normal or elevated (see Table 3). Unlike AAE, C1q levels are normal in HAE. ACE-inhibitor-induced angioedema should be considered if complement studies are normal and the patients is currently using ACE inhibitor therapy. Patients with AAE should also be evaluated for an underlying B-cell lymphoproliferative disorder at the time of diagnosis [18].

## Treatment

The treatment of idiopathic angioedema is similar to that of urticaria. The condition responds well to prophylactic antihistamines. In some individuals, corticosteroids are required. Alcohol and NSAIDs can exacerbate this condition and, therefore, avoidance is advised.

The management of HAE and AAE involves both prophylactic strategies to prevent attacks of angioedema as well as pharmacologic interventions for the treatment of acute attacks (see Table 4). Given the risk of fatal asphyxiation with laryngeal involvement, patient education regarding the treatment of acute attacks is imperative; patients should be instructed to proceed immediately to the emergency department should laryngeal swelling develop. Since HAE and AAE are rare disorders, some emergency department personnel are not familiar with the treatment of these conditions. Therefore, patients are encouraged to carry wallet cards (templates available at: http://www.haecanada.com/m.php?p=edownloads) that briefly explain the patient's diagnosis, outlines the indicated treatment for acute attacks, and provide contact information for the supervising clinician [18]. In patients with AAE, treatment of the underlying lymphoproliferative disorder is also important.

**Table 4:** Overview of therapeutic interventions for HAE and AAE.

Prophylaxis	Acute attacks
• Trigger avoidance – Mild trauma – Anxiety/stress – *H. pylori* infection – ACE inhibitors – Estrogen-containing medications • Attenuated androgens – Danazol – Stanozolol • Tranexamic acid • C1 inhibitor replacement therapy	• C1 inhibitor replacement therapy • Ecallantide (kallikrein inhibitor) • Icatibant (bradykinin receptor blocker)

ACE: angiotensin-converting enzyme

### Prophylactic treatment

Prophylactic therapy should be considered in patients who experience more than one severe attack per month, or if treatment for acute episodes is not sufficiently effective or is not available. Therapeutic options include: trigger avoidance, attenuated androgens, tranexamic acid, and plasma-derived C1 inhibitor replacement therapy [18,24].

Factors triggering acute attacks of AAE and HAE vary but often include: mild trauma to the face (particularly dental trauma), stress/anxiety, *H. pylori* infection, menstruation, and the use of estrogen-containing medications (e.g., hormone replacement therapy and contraceptives) and ACE inhibitors. Whenever possible, these triggers should be avoided. Recognition and prompt treatment of oral and dental infections, and screening for and eradication of *H. pylori* infection may be warranted in some cases [18].

Attenuated androgens, such as danazol and stanozolol, increase C4 and C1 inhibitor levels and are effective for both the short- and long-term prophylaxis of HAE and AAE. Although generally well-tolerated by most patients, adverse effects with long-term androgen administration may include virilization, abnormalities in serum transaminases, menstrual irregularities, hair growth, decreased libido, weight gain, vasomotor symptoms, lipid abnormalities, and depression. Therefore, the lowest effective dose should be utilized (maximum long-term recommended doses are 200 mg daily for danazol and 2 mg daily for stanozolol), and the patient’s CBC, liver enzymes and lipid profile should be monitored regularly (e.g., every 6 months) while on therapy. Contraindications to androgen therapy include: pregnancy, lactation, cancer, hepatitis, and childhood [18].

The antifibrinolytic agent, tranexamic acid, has also been shown to be effective for the prophylactic treatment of HAE and AAE. Evidence suggests that it may be less effective than androgen therapy in patients with HAE, but more effective in AAE [18]. Tranexamic acid is well-tolerated and is generally preferred for long-term prophylaxis in pregnant women, children, and patients who do not tolerate androgens. The most common side effect is dyspepsia, which can be reduced by taking the drug with food [18].

Regular intravenous injections of plasma-derived C1 inhibitor replacement therapy are also effective for both short- and long-term prophylaxis. These injections can usually be administered at home by the patient or caregiver. The current recommended dose is 20 units/kg; patients with AAE may require higher doses. Since C1 inhibitor replacement therapy is a blood product, annual recipient hemovigilance and vein-to-vein tracking are essential [18].

### Treatment of acute attacks

First-line therapies for the treatment of severe acute attacks of HAE and AAE include: C1 inhibitor replacement therapy, ecallantide and icatibant [18]. C1 inhibitor replacement therapy is the most well-studied first-line therapy; it is administered “on-demand” at the first sign of an attack. However, some patients with AAE may become non-responsive to this treatment over time; in these patients the use of ecallantide and icatibant should be considered [24].

Ecallantide is an inhibitor of plasma kallikrein (the enzyme that releases bradykinin, the primary mediator of angioedema). It has recently been approved by the Food and Drug Administration (FDA) in the United States for the treatment of acute angioedema attacks in patients with HAE. The usual recommended dose is 30 mg subcutaneously (adults). Although the side effects of ecallantide are generally mild (i.e., injection-site reactions, headache, nausea, fatigue, diarrhea), this therapy has been associated with rare instances of allergic reactions and anaphylaxis. Therefore, it should only be administered by a clinician in a medical setting equipped to manage anaphylaxis and severe angioedema [18].

Icatibant is a bradykinin receptor blocker that has been approved in the European Union for the treatment of acute attacks of HAE. The usual recommended dose for adults is 30 mg subcutaneously; pediatric experience with this agent is still pending. The most common side effects of icatibant are mild and transient injection site reactions. Other, less common side effects include: nausea, gastrointestinal upset, asthenia, dizziness, and headache [18].

## Conclusions

Urticaria is a common disorder that often presents with angioedema. It is generally classified as acute (lesions occurring for < 6 weeks), chronic (lesions occurring for > 6 weeks) and physical (lesions result from a physical stimulus). The disorder can usually be diagnosed on the basis of clinical presentation and history, however, diagnostic tests may be helpful for confirming the diagnosis. Second-generation, non-sedating H1-receptor antihistamines represent the mainstay of therapy for both acute and chronic urticaria; first-generation sedating antihistamines may be used as adjunctive therapy in patients with nocturnal symptoms. For severe, refractory chronic urticaria, short courses of oral corticosteroids and certain immunosuppressant and immunomodulatory therapies may be beneficial.

Angioedema can occur in the absence of urticaria. The more common causes are ACE inhibitor-induced angioedema and idiopathic angioedema. Rare, but life-threatening causes are HAE or AAE. The work-up and management of HAE and AAE varies considerably from that of angioedema associated with urticaria. Although the angioedema associated with these disorders is often self-limited, laryngeal involvement can lead to fatal asphyxiation. Patients with these disorders demonstrate characteristic abnormalities in certain complement levels and, therefore, diagnostic testing of patients with suspected HAE or AAE should include assessment of C4, C1q and CI inhibitor function and antigenic levels. HAE should be considered in patients with an early age of onset and a family history of the disorder; in patients with AAE, there is no family history and age of onset is usually later. Therapeutic options for the prophylaxis of HAE and AAE include: trigger avoidance, attenuated androgens, tranexamic acid, and plasma-derived C1 inhibitor replacement therapy. First-line therapies for the treatment of acute attacks include: C1 inhibitor replacement therapy, ecallantide and icatibant.

## Key take-home messages

• Urticaria is a common disorder characterized by recurrent, pruritic (itchy) lesions with pale centers (wheals) that usually subside within 48 hours; it is often associated with angioedema.

• Mast cells are the primary effector cells in urticaria.

• Urticaria is classified as acute (lesions for < 6 weeks), chronic (lesions > 6 weeks), or physical.

• The diagnosis of urticaria, with or without angioedema, is based primarily on a thorough clinical history; however, diagnostic tests may be helpful in some instances.

• Second-generation, non-sedating H1-receptor antihistamines are the mainstay of therapy for urticaria. Oral corticosteroids and various immunomodulatory/immunosuppressive therapies may also be used for more severe, chronic cases.

• Angioedema can occur in the absence of urticaria, with ACE inhibitor-induced and idiopathic angioedema being the most common causes.

• ACE inhibitors should be discontinued in any individual who presents with angioedema as this condition is associated with life-threatening upper airway angioedema.

• Idiopathic angioedema responds well to prophylactic antihistamines, however, oral corticosteroids may be required in some cases.

• HAE and AAE are rare disorders also characterized by angioedema in the absence of urticaria; they result from a deficiency or dysfunction of the C1 inhibitor (a plasma protease inhibitor that regulates several proinflammatory pathways), and are associated with life threatening upper airway swelling.

• Diagnosis of HAE and AAE should include the assessment of C4, C1q, C1 inhibitor function and antigenic levels.

• The management of these disorders involves both prophylactic strategies to prevent attacks of angioedema (trigger avoidance, attenuated androgens, tranexamic acid, and plasma-derived C1 inhibitor replacement therapy) as well as pharmacological interventions for the treatment of acute attacks (C1 inhibitor replacement therapy, ecallantide and icatibant).

## Competing interests

Dr. Amin Kanani has received consulting fees and honoraria for continuing education from Scherring, GlaxoSmithKline, King Pharma, Merck Frosst, Novartis, CSL Behring and Talecris Biotherapeutics.

Dr. Robert Schellenberg is past president of the Canadian Society of Allergy & Clinical Immunology, a member of the Medical Advisory Board of the British Columbia Lung Association, the National Lung Health Steering Committee and the Scientific Advisory Committee for Respiratory and Allergy Therapy of Health Canada. He is also a member of the Data Safety Monitoring Committee for Asthma Treatment. Dr. Schellenberg has received consulting fees and honoraria for continuing education and participation in advisory committees from GlaxoSmithKline, Merck Frosst, Novartis, Talecris, Bayer Biologics, and CSL Behring.

Dr. Richard Warrington is the past president of the Canadian Society of Allergy & Clinical Immunology and Editor-in-Chief of *Allergy*, *Asthma & Clinical Immunology.* He has received consulting fees and honoraria from Nycomed, CSL Behring and Talecris.
